# Requirement of Rab5 GTPase during heat stress-induced endocytosis in yeast

**DOI:** 10.1016/j.jbc.2024.107553

**Published:** 2024-07-11

**Authors:** Makoto Nagano, Hiroki Shimamura, Junko Y. Toshima, Jiro Toshima

**Affiliations:** 1 Department of Biological Science and Technology, Tokyo University of Science, Tokyo, Japan; 2 Research Center for Drug and Vaccine Development, National Institute of Infectious Diseases, Tokyo, Japan; 3 School of Health Science, Tokyo University of Technology, Tokyo, Japan

**Keywords:** endocytosis, endosome, membrane trafficking, small GTPase, yeast, heat stress, plasma membrane quality control, Rab5

## Abstract

The plasma membrane (PM) is constantly exposed to various stresses from the extracellular environment, such as heat and oxidative stress. These stresses often cause the denaturation of membrane proteins and destabilize PM integrity, which is essential for normal cell viability and function. For maintenance of PM integrity, most eukaryotic cells have the PM quality control (PMQC) system, which removes damaged membrane proteins by endocytosis. Removal of damaged proteins from the PM by ubiquitin-mediated endocytosis is a key mechanism for the maintenance of PM integrity, but the importance of the early endosome in the PMQC system is still not well understood. Here we show that key proteins in early/sorting endosome function, Vps21p (yeast Rab5), Vps15p (phosphatidylinositol-3 kinase subunit), and Vps3p/8p (CORVET complex subunits), are involved in maintaining PM integrity. We found that Vps21p-enriched endosomes change the localization in the vicinity of the PM in response to heat stress and then rapidly fuse and form the enlarged compartments to efficiently transport Can1p to the vacuole. Additionally, we show that the deubiquitinating enzyme Doa4p is also involved in the PM integrity and its deletion causes the mislocalization of Vps21p to the vacuolar lumen. Interestingly, in cells lacking Doa4p or Vps21p, the amounts of free ubiquitin are decreased, and overexpression of ubiquitin restored defective cargo internalization in *vps9*Δ cells, suggesting that defective PM integrity in *vps9*Δ cells is caused by lack of free ubiquitin.

Maintenance of plasma membrane (PM) integrity is important for normal cell viability and function (1). The PM is constantly exposed to stress from the extracellular environment (2), and these stresses, such as heat and oxidative stress, cause misfolding or denaturation of cell surface proteins, resulting in the accumulation of damaged proteins at the PM and impairment of PM integrity (1). To remove such damaged proteins, eukaryotic cells have a protein quality control system operating on the PM (PMQC) (3). This system is highly conserved from yeast to higher organisms, and the budding yeast is a valuable model organism for investigating the mechanism (4).

The PMQC system plays a key role in maintaining PM integrity. In yeast proteins damaged by heat stress on the PM are ubiquitinated by E3 ubiquitin ligase Rsp5p and arrestin-related trafficking adaptors, and thereby removed from the PM by endocytosis (4). Dysfunction of this ubiquitination system leads to an increase of toxic misfolded proteins, which compromise the barrier function of the PM, thus reducing cell stress tolerance (4). Endocytosed proteins from the PM are transported to the lysosome/vacuole *via* endosomes and degraded. In addition to PMQC, along the endocytic pathway, two PQC systems–endosome quality control (EQC) and lysosome quality control (LQC)–have been reported (3, 5, 6). EQC works to ubiquitinate damaged proteins that have passed PMQC without ubiquitination and direct them into the intraluminal vesicles of the multivesicular body (MVB). On the other hand, LQC works to ubiquitinate misfolded proteins that have been mistargeted onto the vacuolar membrane, thereby facilitating intravacuolar degradation of the proteins (3, 5).

While the EQC and LQC systems are essential for the efficient removal of toxic misfolded proteins at the endo-lysosomal trafficking pathway, the PMQC system, which controls cargo internalization, is reported to be especially important for the maintenance of PM integrity under heat stress conditions (4). Since heat stress affects the entire PM and leads to the widespread generation of the toxic misfolded proteins (4), a mechanism that efficiently internalizes large amounts of damaged proteins into early endosomes should exist. However, the role of the early endosome in the PMQC system under heat stress conditions is not well understood. A recent study using yeast has revealed that a specific *trans*-Golgi network (TGN) sub-compartment (termed the Tlg2p-residing compartment), which includes a yeast syntaxin homolog, Tlg2p, functions as the early endosomal compartment, sorting endocytic cargos to the endo-lysosomal pathway or the recycling pathway (7). Thus, in yeast endo-lysosomal pathway endocytic cargos seem to be first transported to the Tlg2p-residing compartment and then transported to the Vps21p-enriched endosome (7, 8), although it has been unclear whether the Vps21p-enriched endosome interacts directly with endocytic vesicles.

In the present study, we examined the role of early-to-late endosomal transport and maturation in the PMQC system. We were able to show that key proteins for early endosome function, Vps21p (yeast Rab5), Vps15p (phosphatidylinositol-3 kinase subunit), and Vps3p/8p (CORVET complex subunits), are involved in the maintenance of PM integrity. Vps21p plays two distinct roles in the PMQC system under heat stress: efficient incorporation of damaged proteins from the PM into the endosomal compartment and rapid transport of endocytosed proteins into the prevacuolar compartment. We also clarified that Doa4p, a deubiquitinating enzyme, is involved in PM integrity and its deletion causes the mislocalization of Vps21p to the vacuolar lumen. Surprisingly, cells lacking Vps21p have significantly reduced levels of free mono-ubiquitin, and therefore the present findings suggest that Vps21p-enriched endosomes play crucial roles in the degradation of the damaged cell surface proteins in the PMQC system.

## Results

### Vps21p-mediated endosomal trafficking is required for the maintenance of PM integrity

**Figure 1:**
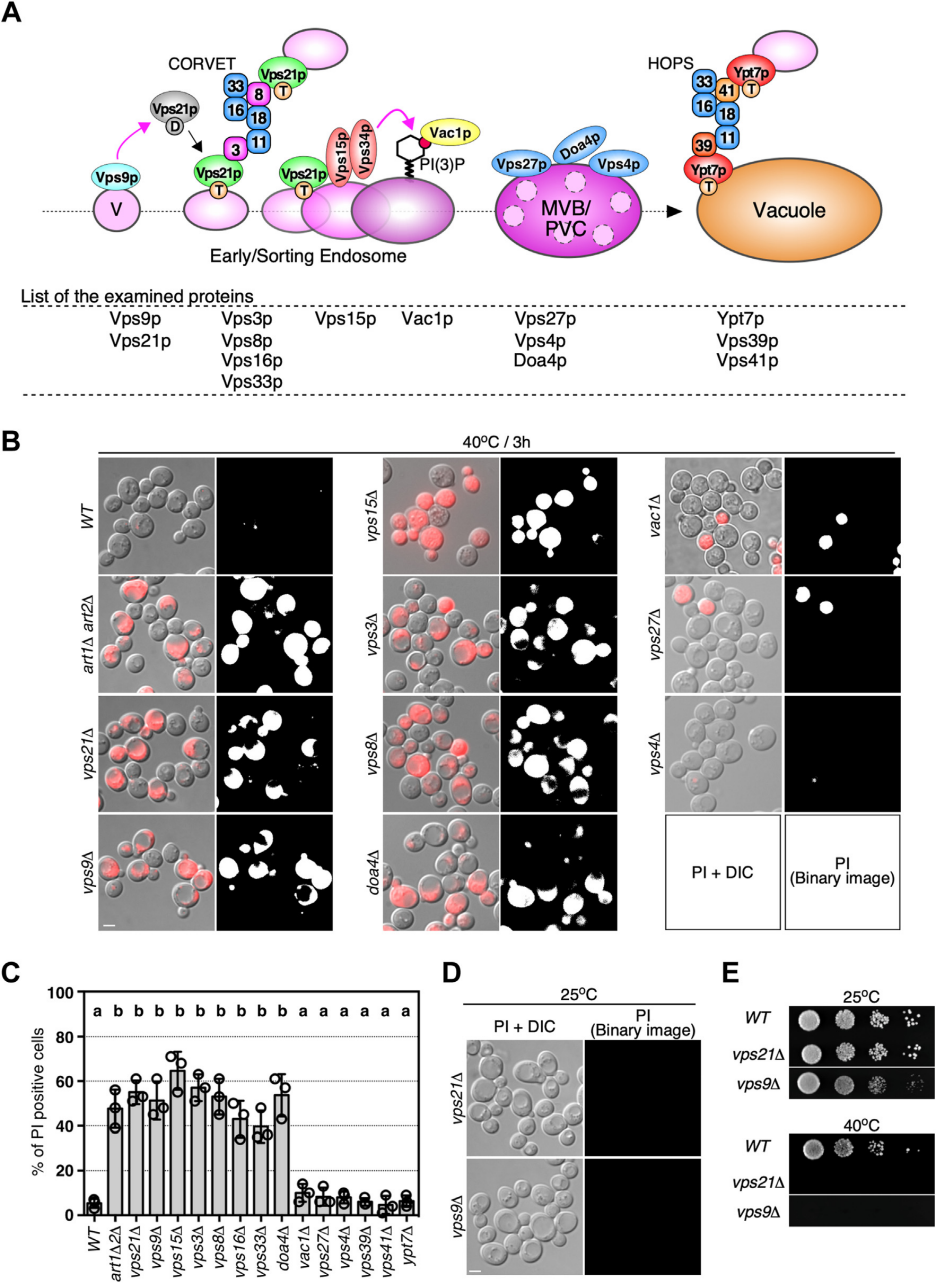
**Vps21p and the effector proteins are required for maintaining the plasma membrane integrity under heat stress condition.***A*, schematic diagram of endosome formation/maturation mediated by Vps21p and its effector proteins. *B*, propidium iodide (PI) staining of wild-type and mutant cells under heat stress conditions. The cells were grown to early-mid logarithmic phase at 25 °C, then cultured at 40 °C for 3 h, and stained with PI. Fluorescence images overlaid with DIC images were shown in the left panels. For quantifying the percentage of PI-positive cells, fluorescence images were converted to binary images as shown in the right panels. *C*, the percentage of PI-positive cells in wild-type and mutant cells. *D*, PI staining of wild-type and mutant cells under non-stress conditions. The *vps21*Δ or *vps9*Δ cells were analyzed in the same way as (*B*). *E*, growth phenotype of the cells under heat stress condition. A dilution series of *vps21*Δ or *vps9*Δ was spotted on YPD plates and incubated at the indicated temperature for 2 (25 °C) or 6 (40 °C) days. Data show the mean ± SEM of three independent experiments in which 100 cells were scored per experiment (*C*). Different letters indicate significant difference at *p* < 0.05, one-way ANOVA with Tukey’s *post hoc* test (*C*). Scale bar in all panels, 2.5 μm.

Previous studies have shown that cells in which the PMQC system does not function properly have defective PM integrity (2, 4, 9). One of the mechanisms required for PMQC is ubiquitin-mediated endocytosis, for which Rsp5-ART ubiquitin ligase recognizes aberrant cell surface proteins, ubiquitinates them, and mediates their degradation in the vacuole *via* the endocytic pathway (4). However, as the importance of endosomes in the PMQC system has not been well understood, we examined the involvement of the endosome in this system. To identify the endosomal proteins required for PMQC, we selected several proteins that are involved in different steps of endosomal trafficking and examined the PM integrity of cells lacking each of the individual genes (Fig. 1*A*). To examine the proportion of cells that showed loss of PM integrity, we exposed cells to high temperatures (40 °C) for 3 h and then stained them using propidium iodide (PI), which is a PM-impermeant DNA binding dye (4). Consistent with the previous study (4), ∼ 47.7% of *art1*Δ *art2*Δ double mutant cells showed defective PM integrity after heat stress, whereas wild-type cells showed only a slight defect (∼5.3%) (Fig. 1, *B* and *C*). Interestingly, we found that deletion of *VPS21* or *VPS9*, encoding yeast Rab5 homolog Vps21p or the specific GEF Vps9p, impaired PM integrity during heat stress, although no PM integrity defect was evident at 25 °C (Fig. 1, *B*–*D*). We also examined the requirement of Ypt53p, which is a stress-induced isoform of Vps21p (10, 11), but the *ypt53*Δ mutant exhibited a negligible defect of PM integrity (Fig. S1, *A* and *B*). It has been shown that the mutants with defective PM integrity generally exhibit a temperature-sensitive growth defect (4), and the cells lacking *VPS21* and *VPS9* were also inviable at 40 °C (Fig. 1*E*). Deletion of the gene encoding Vps3p/Vps8p (subunits of the CORVET complex) (12) or Vps15p (the regulatory subunit of the PtdIns 3-kinase complex) (13), which bind to Vps21p, also resulted in significant impairment of PM integrity (Fig. 1, *B* and *C*), suggesting the involvement of Vps21p in PM maintenance. The cells lacking *VPS16* or *VPS33*, which encode a common core subunit of the CORVET and the HOPS complexes, also impaired PM integrity (Fig. S1*C*). On the other hand, as suggested previously (4), deletion of endosomal proteins that function in the late stage of endosomal sorting, such as Vac1p, or MVB formation regulated by the ESCRT complex, such as Vps27p and Vps4p exhibited only negligibly defective PM integrity (Fig. 1, *B* and *C*). Consistent with this, yeast Rab7 homolog Ypt7p and the Vps39p/41p (subunits of the HOPS complex) that also function in the late stage of endosomal trafficking were dispensable for the PM integrity (Fig. S1*C*). Accordingly, these results suggest that the regulation of endosomal trafficking by Vps21p and its effector proteins play a critical role in PMQC *via* the endocytic pathway.

### The Vps21p-enriched endosome is required for the removal of denatured cell surface proteins resulting from heat stress condition

**Figure 2:**
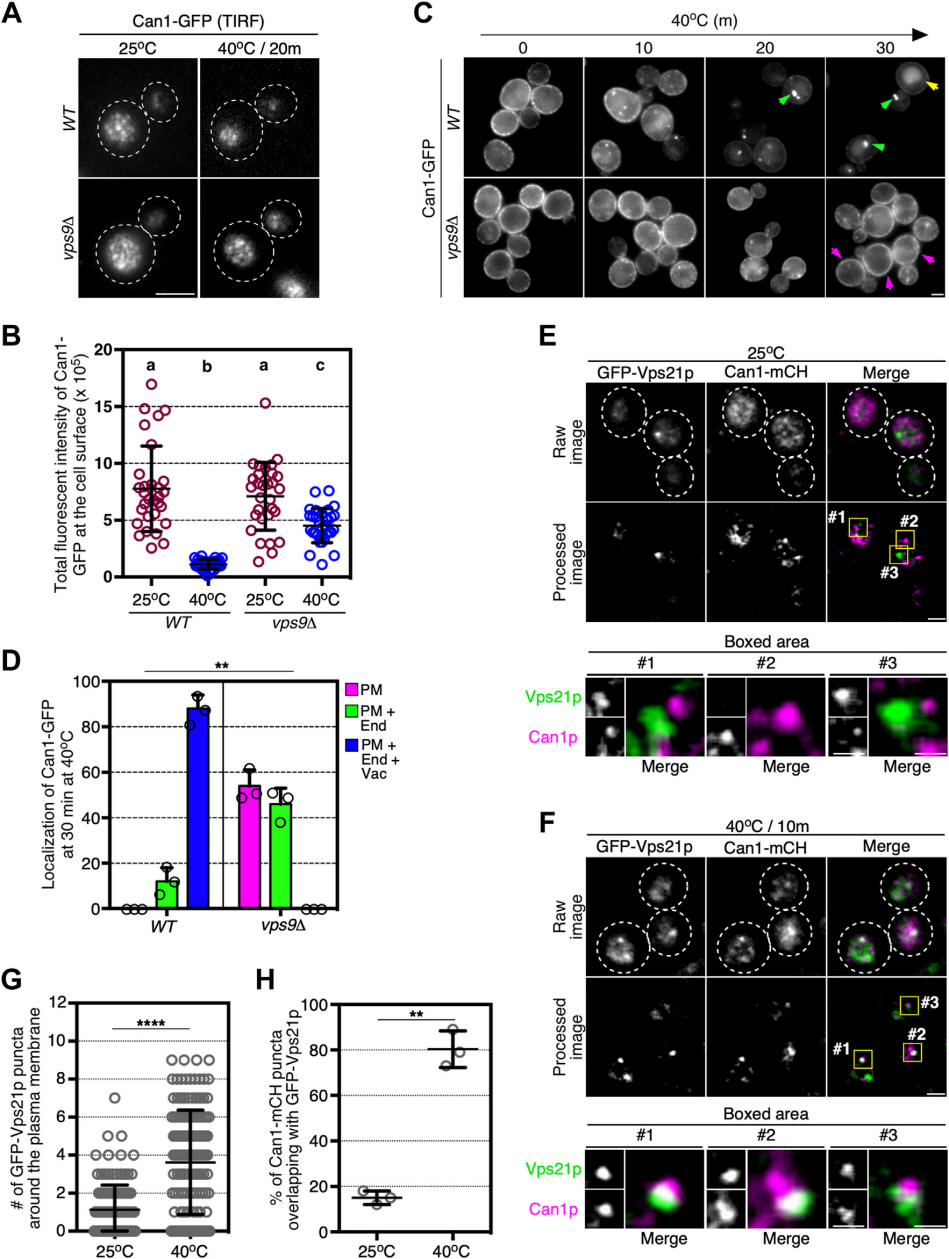
**Vps21p promotes internalization of the arginine transporter Can1p under heat stress.***A*, effect of *VPS9* deletion on the endocytic clearance of Can1-GFP from cell surface during heat stress. Cells expressing Can1-GFP from the endogenous locus were grown to early-mid logarithmic phase at 25 °C (*left panels*), then cultured at 40 °C for 20 min (*right panels*), and analyzed by total internal reflection fluorescence (TIRF) microscopy. *B*, quantification of Can1-GFP fluorescence intensity at the cell surface. The total fluorescent intensity of Can1-GFP per cell was quantified and plotted as individual spots on the graph. *C*, The spatiotemporal localization of Can1-GFP in wild-type or *vps9*Δ cells under heat stress conditions. Cells were grown to early-mid logarithmic phase at 25 °C, then cultured at 40 °C for the indicated time. *Green* and *yellow arrows* indicate the localization of Can1-GFP at endosome-like compartments and in vacuole (upper panels). Magenta arrows indicate that Can1-GFP resides at the plasma membrane even after heat stress (lower panels). *D*, quantification of Can1-GFP localization in wild-type or *vps9*Δ cells after being cultured at 40 °C for 30 min. Plasma membrane only (PM), plasma membrane and endosome (PM + End), and plasma membrane, endosome and vacuole (PM + End + Vac). *E* and *F*, localization of Can1-mCherry and GFP-Vps21p at the cell surface under non-stress (*E*) or heat stress condition. Cells expressing Can1-mCherry and GFP-Vps21p were grown to early-mid logarithmic phase at 25 °C (*E*), then cultured at 40 °C for 10 min (*F*), and analyzed by TIRF microscopy. The fluorescent images (Raw image) were processed to visualize the punctate localization of Can1-mCherry and GFP-Vps21p at the cell surface (Processed image) by sequential filtering using top-hat and bilateral filters bundled in the Image J FIJI software package as described in the Experimental procedures section. Higher magnification view of the boxed area in the processed images are displayed in the lower panels. *G*, quantification of the number of GFP-Vps21p puncta at 25 °C and 40 °C. *H*, quantification of Can1-mCherry puncta overlapping with GFP-Vps21p at the surface of cells. Data show the mean ± SD with 30 cells (*B*) or 150 puncta (*G*) from three independent experiments or the mean ± SEM of three independent experiments in which 100 cells (*D*) or 100 puncta (*H*) were scored per experiment. Different letters indicate significant difference at *p* < 0.05, one-way ANOVA with Tukey’s *post hoc* test (*B*). ∗∗*p* < 0.01, ∗∗∗∗*p* < 0.0001, *chi*-square test for trend (*D*), two-tailed unpaired *t* test with Welch’s correction (*G* and *H*). Scale bar in panels in *A*, *C*, *E* (*upper*) and *F* (*upper*), 2.5 μm, or *E* (*lower*) and *F* (*lower*), 1.0 μm.

We next focused on the role of Vps21p in the removal of aberrant cell surface proteins from the PM and examined the effect of *VPS9* deletion on the arginine transporter Can1p, a cell surface protein that is known to be rapidly endocytosed and degraded in the vacuole under heat stress conditions (4). To examine the PM localization of Can1p, we imaged Can1-GFP using total internal reflection fluorescence microscopy (TIRFM). In agreement with the previous observation (4), Can1-GFP was rapidly cleared from the PM (decreased to ∼17%) of wild-type cells in response to heat stress at 40 °C for 20 min (Fig. 2, *A* and *B*). In contrast, in the *vps9*Δ cell, the expression level of Can1-GFP at the PM was similar to that in wild-type cells, but heat stress-induced endocytic internalization was severely blocked, ∼56% of Can1-GFP remaining at the PM after heat stress (Fig. 2, *A* and *B*). This was an unexpected finding because we have previously demonstrated that Vps21p activity is not required for an internalization step of endocytic cargo (14). After heat stress, middle focal plane observation of wild-type cells by epi-fluorescence microscopy revealed Can1-GFP to be localized at the small intracellular vesicles as well as at the PM at 10 min, later becoming localized at the larger endosomal compartments at 20 min, and accumulating in the vacuole at 30 min (Fig. 2*C*). In *vps9*Δ cells, localization of Can1-GFP was considerably increased at the PM, and little vacuole localization was evident even at 30 min after heat stress (Fig. 2, *C* and *D*). This increased PM localization of Can1p could be attributable to either inhibition of endocytosis or enhanced recycling of endocytosed Can1p to the PM. Since deletion of Rcy1p, which regulates the recycling pathway through the TGN, causes accumulation of cargo at the TGN (15), we constructed a double mutant lacking Vps9p and Rcy1p to investigate whether Can1p recycling is enhanced in *vps9*Δ cells. In the *vps9*Δ *rcy1*Δ double mutant cells, we observed that Can1-GFP still accumulated at the PM under heat stress conditions (Fig. S2, *A* and *B*). Therefore, it appears that the increased localization of Can1p at the PM in *vps9*Δ mutants is due to defective internalization of Can1p.

We then investigated the localization of Vps21p during heat stress-induced endocytosis of Can1p. To clearly detect the localization of Can1-GFP at the PM, we reduced the fluorescence background of Can1-GFP using the top-hat (16) and bilateral (17) filters bundled in the Image J FIJI software package (18). This image processing allowed us to more accurately determine the location of Can1-GFP at the PM (Fig. 2*E*). Two color live imaging using Can1-mCherry and GFP-Vps21p demonstrated that the PM localization of Vps21p was clearly increased under heat stress at 40 °C for 10 min, relative to normal conditions at 25 °C (Fig. 2, *E* and *F*). Quantitative analysis revealed that the average number of GFP-Vps21p puncta observed around the PM by TIRFM was ∼1.1 at 25, whereas at 40 °C the number increased to ∼3.6 (Fig. 2*G*). We also found that Can1p signals overlapping with Vps21p increased from ∼15.0% to ∼80.3% after heat stress for 10 min (Fig. 2*H*). To confirm that the PM localization of Vps21p under heat stress is dependent on the denaturation of PM proteins, we examined the localization of GFP-Vps21p in the presence of glycerol, which is known to function as a chemical chaperon and protect protein folding (4, 19). Consistent with the previous observation, heat stress-induced endocytosis of Can1-GFP was suppressed by glycerol treatment (Fig. S2*C*). TIRFM imaging of glycerol-treated cells revealed that the PM localization of Vps21p was remarkedly decreased in the presence of glycerol (Fig. S2, *D* and *E*). We further examined whether PM localization of Vps21p would also occur in arginine-induced endocytosis of Can1p, which is ubiquitination-dependent under non-stress conditions (20). In the presence of arginine, Can1p was rapidly endocytosed in both wild-type and *vps9*Δ cells, although transport of Can1p to the vacuole was blocked in *vps9*Δ cells (Fig. S2, *F* and *G*). Thus, Vps21p-enriched compartments seemed to change the localization in the vicinity of the PM to facilitate endocytic internalization of Can1p specifically under heat stress.

### Heat stress causes the formation of enlarged Vps21p-enriched compartments

**Figure 3:**
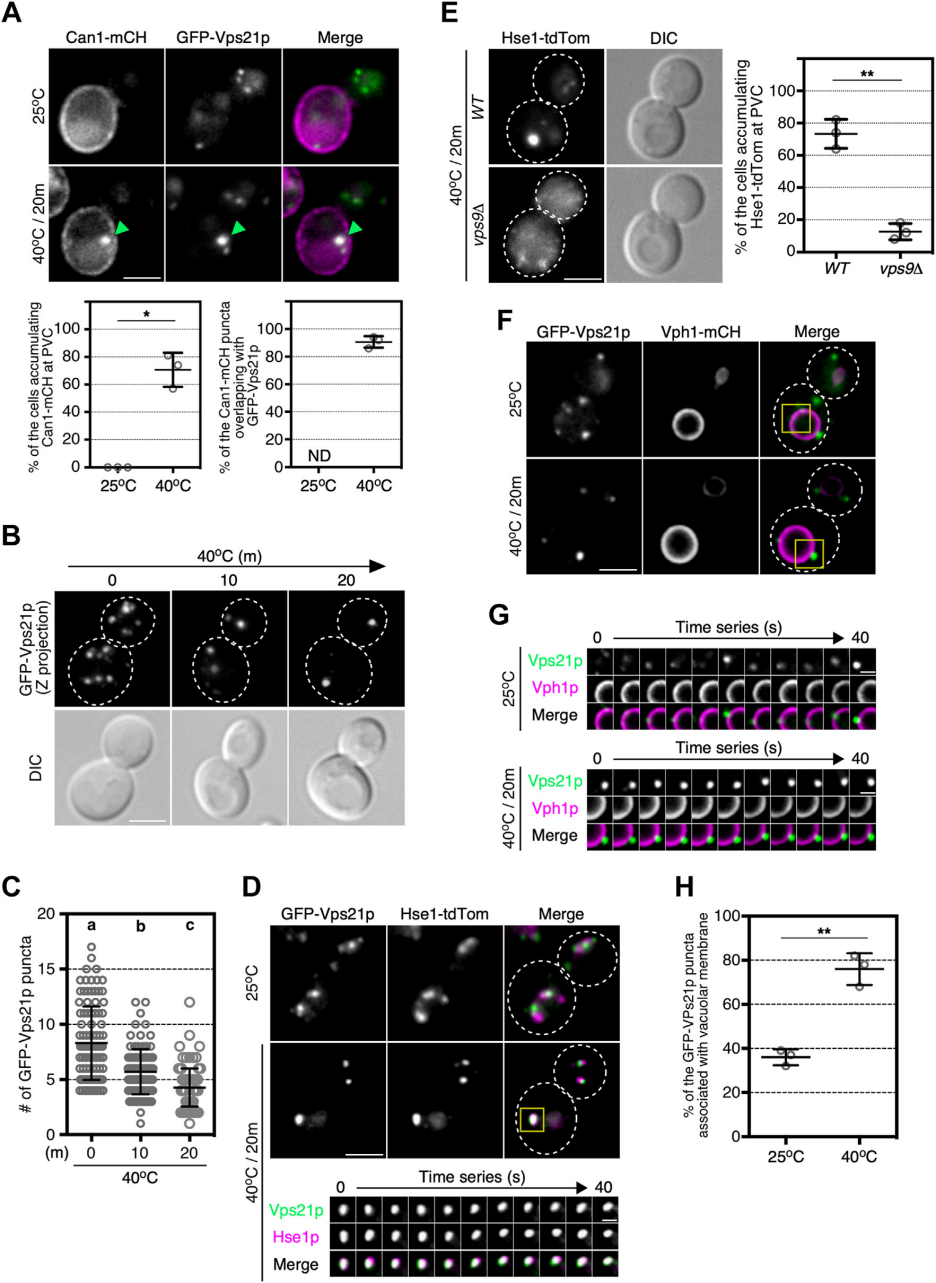
**Dynamics of Vps21p-enriched compartments under heat stress conditions.***A*, localization of Can1-mCherry and GFP-Vps21p under non-stress or heat stress conditions. Cells expressing Can1-mCherry and GFP-Vps21p were grown to early-mid logarithmic phase at 25 °C, then cultured at 40 °C for 20 min, and analyzed by epifluorescence microscopy. *Green arrowheads* indicate the example of Can1-mCherry puncta overlapping with GFP-Vps21p. Graphs represent the percentage of the cells exhibiting Can1p localization at the prevacuolar compartment (PVC) (*left*) and the percentage of Can1-mCherry puncta overlapping with GFP-Vps21p (*right*). *B*, maximum-intensity projections of z-stacks of the cells expressing GFP-Vps21p. The z series was acquired through the entire cell at 0.4 μm intervals. *C*, quantification of the number of GFP-Vps21p puncta under heat stress conditions. *D*, localization of Hse1-tdTomato and GFP-Vps21p under heat stress conditions. Localization of Hse1-tdTomato and GFP-Vps21p was analyzed in the same way as A. Time series of the region in the boxed area in the middle panel are shown in the *lower panels*. *E*, localization of Hse1-tdTomato in wild-type or *vps9*Δ cells under heat stress condition. Localization of Hse1-tdTomato was analyzed in the same way as A. Graphs represent the percentage of the cells exhibiting Hse1p localization at the PVC. *F*, localization of GFP-Vps21p around the vacuole under heat stress conditions. Localization of GFP-Vps21p and Vph1-mCherry was analyzed in the same way as *A*. *G*, dynamics of GFP-Vps21p around the vacuole under heat stress conditions. Time series of the boxed area in *F* is shown. *H*, percentage of the association of GFP-Vps21p puncta with vacuolar membrane. Percentages of GFP-Vps21p puncta associating with vacuolar membrane at 25 °C and 40 °C. Data show the mean ± SD with 150 puncta (*C*) from three independent experiments or the mean ± SEM of three independent experiments in which 100 cells (*A*-*left*, *E*) or 100 puncta (*A-right*, *H*) were scored per experiment. Different letters indicate significant difference at *p* < 0.05, one-way ANOVA with Tukey’s *post hoc* test (*C*). ∗*p* < 0.05, ∗∗*p* < 0.01, ND, not datamined, two-tailed unpaired *t* test with Welch’s correction (*A*, *E*, *H*). Scale bar in panels in *A*, *B*, *D* (*upper*), *E* and *F*, 2.5 μm, or *D* (*lower*) and *G*, 1.0 μm.

Since we found that heat stress changes the localization of Vps21p, we next investigated the dynamics of Vps21p during transport of Can1p from the endosome to the vacuole. As shown above, Can1p mostly localized at the PM and did not colocalize with GFP-Vps21p-labeled puncta (∼0%) at 25 °C (Fig. 3*A*), whereas the two were highly co-localized at the larger endosomal compartment proximal to the vacuole (∼90.7%) at 20 min after heat stress (Fig. 3*A*). Interestingly, under heat stress conditions at 40 °C for 20 min, we found that the size of the Vps21p-enriched compartments increased while their number decreased (Fig. 3*A*). To accurately examine the number of Vps21p-enriched compartments, we analyzed their localization by maximum-intensity projection of Z stacks and found that at 25 °C the number of GFP-Vps21p-enriched compartments was ∼8.3 but decreased to ∼5.7 at 10 min and to ∼4.3 at 20 min after heat stress (Fig. 3, *B* and *C*). We investigated whether other proteotoxic stresses (growth in 10% ethanol or 5 mM dithiothreitol (DTT)), which have been reported to induce endocytosis of damaged membrane proteins, would also trigger the formation of enlarged Vps21p-enriched compartments. Similarly to heat stress, ethanol and DTT induced endocytosis of Can1p to the vacuole and formation of enlarged Vps21p-enriched compartments (Fig. S3, *A* and *B*). Co-labeling of the Vps21p-enriched compartments with tdTomato-tagged Hse1p, a subunit of the ESCRT-0 complex that localizes to the early-to-late endosome (14), revealed that Hse1p was also localized at large Vps21p-enriched endosomes and that their colocalization increased (Fig. 3*D*). Thus, it was likely that, under heat stress conditions, only the localization of Vps21p was changed, although endosomal fusion was promoted, resulting in a decrease in the number of endosomes (Fig. 3, *D* and *E*). Since we have previously shown that vesicle transport from the TGN is crucial for Vps21p activation and subsequent endosome formation (8), we speculated that post-Golgi transport is required for the formation of heat stress-induced aberrant Vps21p-enriched compartments. As expected, deletion of Arf1p GTPase or the clathrin adaptors Ent3p/5p, which block(s) vesicle transport from the TGN (8), significantly reduced the formation of enlarged Vps21p-enriched endosomes (Fig. S3, *C* and *D*). In *vps9*Δ cells, localization of Hse1-tdTomato to the enlarged endosomes was significantly decreased by heat stress (Fig. 3*E*), supporting the idea that heat stress promotes endosomal fusion mediated by Vps21p. Thus, these large compartments formed as a result of heat stress appear to be late endosome-like structures, although their dynamics differ largely from the normal late endosome. At 25 °C Vps21p-enriched endosomes moved actively in the cytosol and associated only transiently with the vacuolar membrane labeled by mCherry-tagged Vph1p, a vacuolar ATPase (21), whereas the late endosome-like compartments formed under heat stress were quite immobile and stably associated with the vacuolar membrane (Fig. 3, *F* and *G*). Quantitative analysis revealed that only ∼36% of endosomes were associated with vacuoles at 25 °C, but that ∼76% of the large compartments were associated at 40 °C (Fig. 3*H*). As described above Can1p became localized to these large compartments at 20 min after heat stress, and then moved to the vacuole (Fig. 2*C*). Taken together, these findings suggest that under heat stress, Vps21p-enriched endosomes likely approach the PM to facilitate Can1p internalization, and then rapidly fuse and form the enlarged endosomal compartment to efficiently transport Can1p to the vacuole.

### Maintenance of the free ubiquitin level is crucial for the endocytic clearance of damaged protein

**Figure 4:**
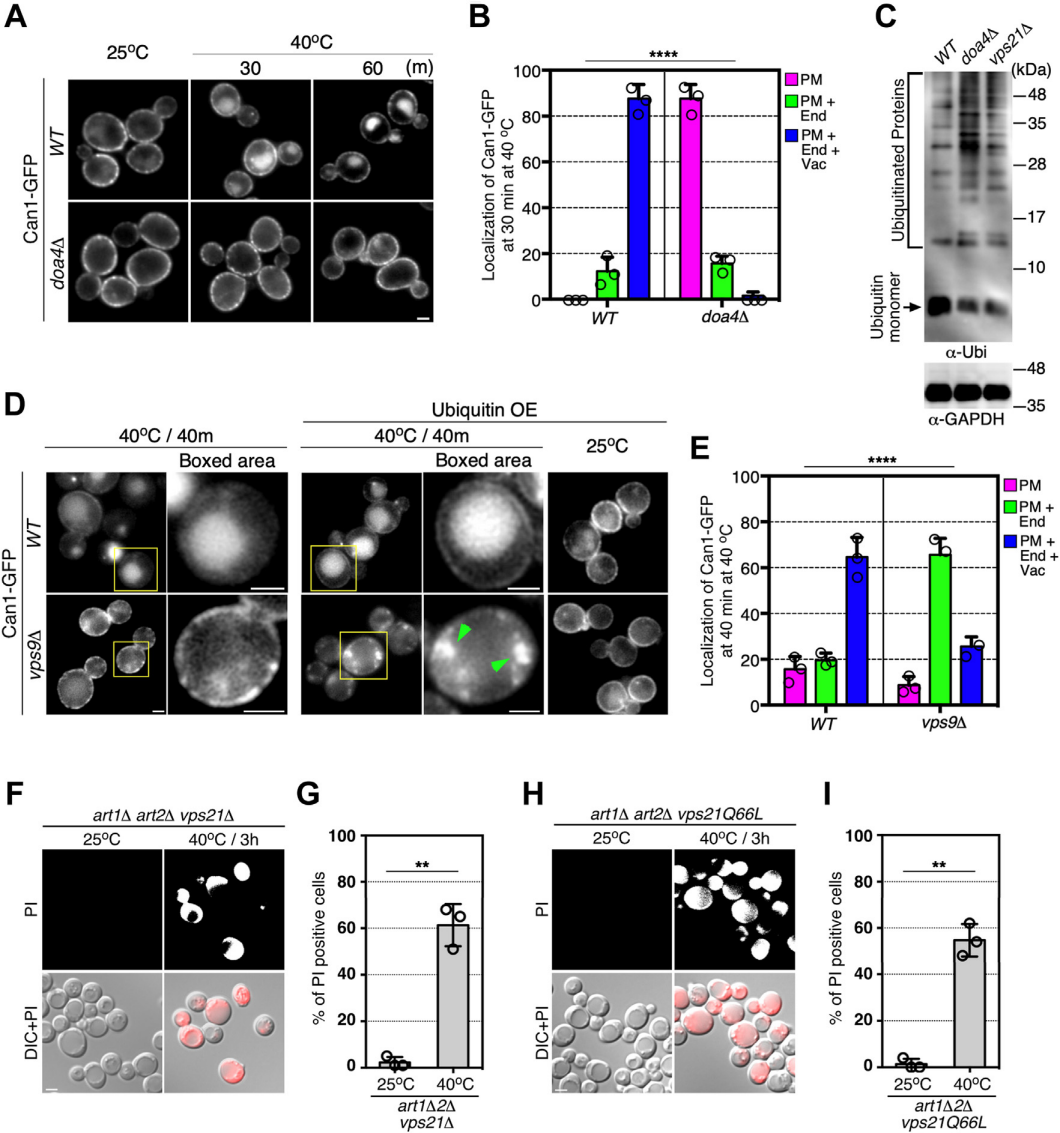
**Role of ubiquitin availability on localization of Can1p under heat stress condition.***A*, the spatio-temporal localization of Can1-GFP in *doa4*Δ cells under heat stress condition. Cells were grown to early-mid logarithmic phase at 25 °C, then cultured at 40 °C for the indicated time, and analyzed by epifluorescence microscopy. *B*, quantification of Can1-GFP localization in the cells after being cultured at 40 °C for 30 min. Plasma membrane only (PM), plasma membrane and endosome (PM + End), and plasma membrane, endosome and vacuole (PM + End + Vac.). *C*, immunoblots showing ubiquitin monomer and ubiquitinated proteins in the extract from wild-type or mutant cells. *D*, localization of Can1-GFP in the *vps9*Δ cells overexpressing ubiquitin under heat stress conditions. Cells transformed by pUB175 were grown to early-to mid-logarithmic phase at 25 °C, treated with 200 μM CuSO_4_ for 2 h, then cultured at 37 °C for 3 h, and analyzed by epifluorescence microscopy. Higher magnification view of the yellow boxed area is displayed at the *right panel*. *Green arrowheads* indicate the localization of Can1-GFP at endosome-like compartments. *E*, quantification of Can1-GFP localization in the cells overexpressing ubiquitin after being cultured at 40 °C for 40 min. Plasma membrane only (PM), plasma membrane and endosome (PM + End), and plasma membrane, endosome and vacuole (PM + End + Vac.). *F and H*, PI staining of the mutant cells under non-stress (25 °C) or heat stress (40 °C) conditions. The cells were analyzed in the same way as Figure 1*B*. *G and I*, the percentage of PI-positive cells in the mutant cells. Data show the mean ± SEM of three independent experiments in which 100 cells (*B*, *E*, *G*, *I*) were scored per experiment. ∗∗∗∗*p* < 0.0001, n.s., not significant, *chi*-square test for trend (*B*, *E*), ∗∗*p* < 0.01, two-tailed unpaired *t* test with Welch’s correction (*G*, *I*). Scale bar in all panels, 2.5 μm. OE, overexpression.

As shown in Figure 1, *B* and *C*, we also found that the deletion of *DOA4*, which encodes a ubiquitin hydrolase that catalyzes protein deubiquitination at the MVB (22, 23), caused a defect of PM integrity in response to heat stress. Furthermore, as was the case in *vps21*Δ cells, heat-induced endocytic internalization of Can1p was severely inhibited in *doa4*Δ cells (Fig. 4, *A* and *B*). As reported previously, we observed that in *doa4*Δ cells the level of free ubiquitin was markedly reduced (Fig. 4*C*) (24, 25). Intriguingly, we found that the mono-ubiquitin level was also decreased in *vps21*Δ cells (Fig. 4*C*), suggesting that increased PM localization of Can1p in *vps9*Δ cells is caused by lack of free ubiquitin. To confirm this possibility, we tested the effect of mono-ubiquitin overexpression on Can1p localization in *vps9*Δ cells. Overexpression of ubiquitin clearly restored Can1p internalization from the PM (Fig. 4, *D* and *E*). Since the free ubiquitin level was decreased in *vps21*Δ cells, we next examined whether deletion of *ART1/2* and *VPS21* would further impairs PM integrity, but found no additive effects was observed (Fig. 4, *F* and *G*). Additionally, expression of the constitutively active form of Vps21p (vps21Q66L) was unable to restore the defective PM integrity in *art1*Δ *art2*Δ mutant under heat stress (Fig. 4, *H* and *I*). These results suggest that Vps21p is required for the maintenance of the free ubiquitin pool, which is necessary for cargo ubiquitination by Rsp5-ART.

### Doa4p is required for Vps21p dissociation from the vacuolar membrane

**Figure 5:**
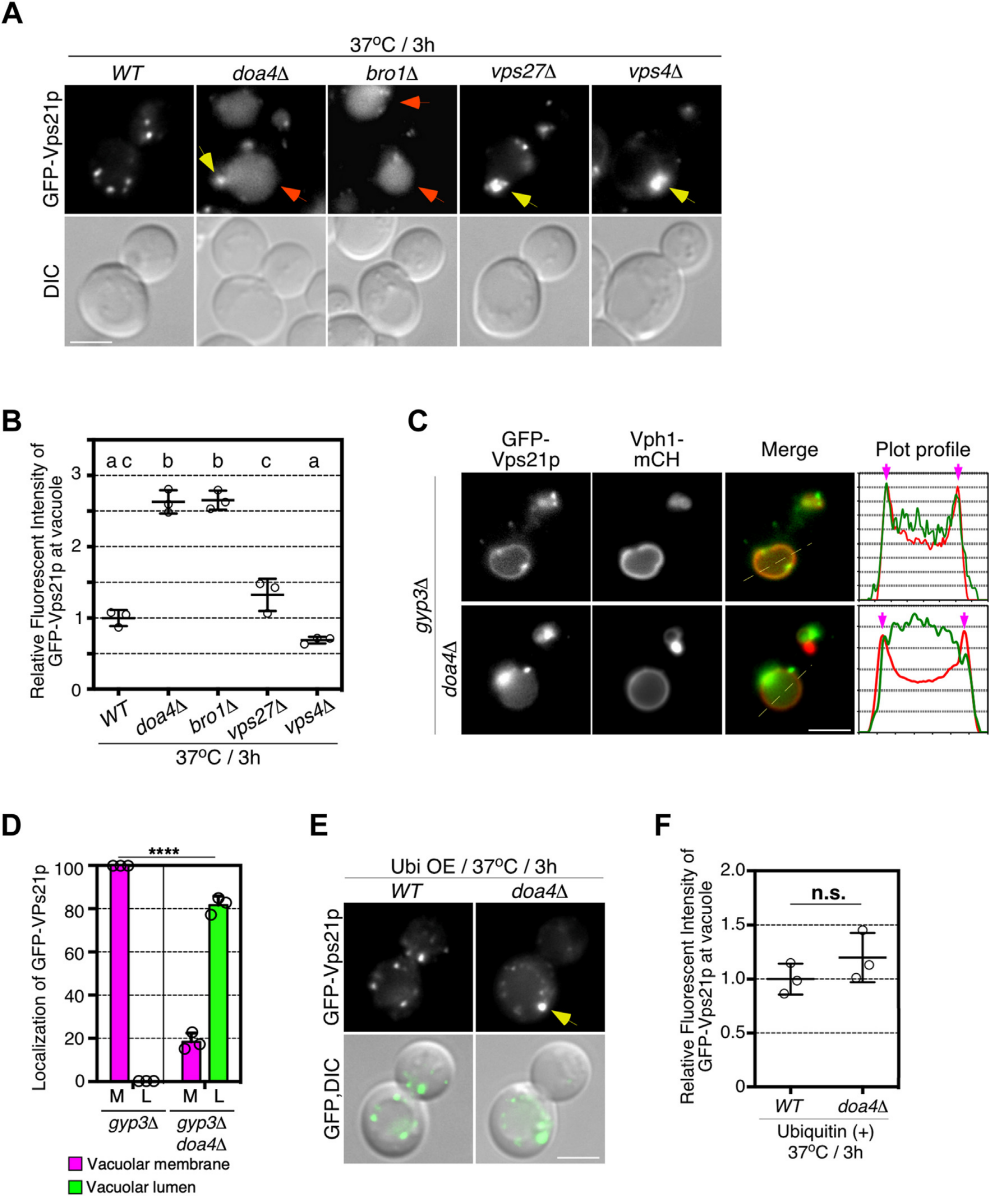
**Role of Doa4p on localization of Vps21p.***A*, localization of GFP-Vps21p in wild-type and mutant cells under heat stress condition. *Yellow* and *red arrows* indicate the localization of GFP-Vps21p at the prevacuolar compartment (PVC) and the vacuolar lumen. *B*, quantification of the fluorescence intensity of GFP-Vps21p at the vacuolar lumen in wild-type and mutant cells. *C*, localization of GFP-Vps21p in *gyp3*Δ or *gyp3*Δ *doa4*Δ cells. Representative fluorescence intensity profiles along the dashed line are indicated in the right graphs. Magenta arrows indicate the positions of the vacuolar membrane. *D*, quantification of GFP-Vps21p localization in the mutants. Vacuolar membrane (M), Vacuolar lumen (L). *E*, localization of GFP-Vps21p in the *doa4*Δ cells overexpressing ubiquitin. Cells transformed by pUB175 were grown to early-to mid-logarithmic phase at 25 °C, treated with 200 μM CuSO_4_ for 2 h, then cultured at 37 °C for 3 h, and analyzed by epifluorescence microscopy. Yellow arrow indicates the localization of GFP-Vps21p at the prevacuolar compartment. *F*, quantification of the fluorescence intensity of GFP-Vps21p at the vacuolar lumen in wild-type and *doa4*Δ cells. Data show the mean ± SEM of three independent experiments in which 100 cells (*B*, *D*, and *F*) were scored per experiment. Different letters indicate significant difference at *p* < 0.05, one-way ANOVA with Tukey’s *post hoc* test (*B*). ∗∗∗∗*p* < 0.0001, n.s., not significant, *chi*-square test for trend (*D*), two-tailed unpaired *t* test with Welch’s correction (*F*). Scale bar in all panels, 2.5 μm. Ubi OE, ubiquitin overexpression.

The recycling of free ubiquitin by Doa4p is required for the role of Vps21p in the PMQC. We examined whether the decreased availability of free ubiquitin pool affects to the intracellular dynamics of Vps21p under heat stress. Interestingly, we found that in *doa4*Δ cells Vps21p was localized in the vacuolar lumen in addition to the prevacuolar compartment at 25 °C (Fig. S4*A*). This vacuolar luminal localization of GFP-Vps21p was not observed in cells lacking other class E *VPS* genes, such as *VPS27* and *VPS4*, which had an enlarged prevacuolar compartment (Fig. S4*A*) (26). The vacuolar localization of GFP-Vps21p was further increased by heat stress at 37 °C (Fig. 5, *A* and *B*). In addition to *doa4*Δ cell, cells lacking Bro1p, which mediates recruitment of Doa4p to the endosomes (27), also exhibited vacuolar lumen localization of Vps21p, but no such localization was observed in *vps27*Δ and *vps4*Δ cells even after heat stress (Fig. 5, *A* and *B*). These observations suggest that the deubiquitination defect of Doa4p cargo(s) causes the mislocalization of Vps21p to the vacuolar lumen. Previous studies have demonstrated that lacking of Vps21p-GAP Gyp3p causes accumulation of Vps21p to the vacuolar membrane, due to being fixed into its GTP-bound form (28, 29). In this mutant, no vacuolar luminal localization of Vps21p was observed, but further deletion of *DOA4* resulted in localization of Vps21p to the vacuolar lumen (Fig. 5*C*). Line scan analysis revealed that most of the Vps21p localized at the vacuolar membrane shifted to the vacuolar lumen (Fig. 5, *C* and *D*). The constitutively activated vps21Q66L mutant also changed the localization into the vacuole in *doa4*Δ cells under the heat stress condition (Fig. S4*B*). In contrast, deletion of *DOA4* did not affect the vacuolar membrane localization of Vph1-mCherry (Fig. 5*C*), suggesting that Vps21p specifically changes its localization in a Doa4p-dependent manner. To examine whether the mis-localization of Vps21p in *doa4*Δ cells was due to reduced availability of free ubiquitin, we tested the effect of mono-ubiquitin overexpression on the Vps21p localization in *doa4*Δ cells. Overexpression of ubiquitin clearly suppressed the vacuolar localization of GFP-Vps21p (Fig. 5, *E* and *F*), suggesting that the recycling of free ubiquitin is important for the maintenance of Vps21p endosomal localization. Thus, it is likely that under the heat stress conditions, free ubiquitin is required to suppress any missorting of Vps21p into the vacuole.

## Discussion

In this study, we investigated the requirement for endosomal proteins in the PMQC system and found that several proteins, including yeast Rab5 Vps21p and its effectors, are involved in the maintenance of PM integrity. Intriguingly, we observed that under heat stress conditions Vps21p-enriched compartments clearly showed increased localization to the PM. Previous studies have demonstrated that deletion of yeast Rab5s had little effect on endocytic cargo internalization from the PM under non-stress conditions (8, 14), suggesting that interaction between the endocytic site and the endosomal compartment is increased specifically under heat stress. Although it has not been clarified whether heat stress promotes the formation and/or internalization of endocytic vesicles or simply increases the uptake of cargos, it has been reported that the Rsp5p-ARTs ubiquitin ligases play a crucial role in the degradation of aberrant cell surface proteins through the endocytic pathway (4, 30). Thus, Vps21p-enriched compartment might increase PM localization by interacting with ubiquitinated cargos promoted by heat stress. Previous studies have shown that cycloheximide also induces Can1p endocytosis by promoting Rsp5p-ART-dependent ubiquitination (31, 32), although Vps21p is not required for internalization of Can1p from the PM in this process. The difference in the role of Vps21p in Can1p internalization under cycloheximide stress and heat stress suggests that these stresses induce Can1p internalization through a different mechanism. Since several chaperones, such as HSP60, HSP70, and HSP90, are known to relocalize to the PM in response to heat stress (33), these chaperones may be involved in the recruitment of Vps21p to the PM under heat stress.

**Figure 6:**
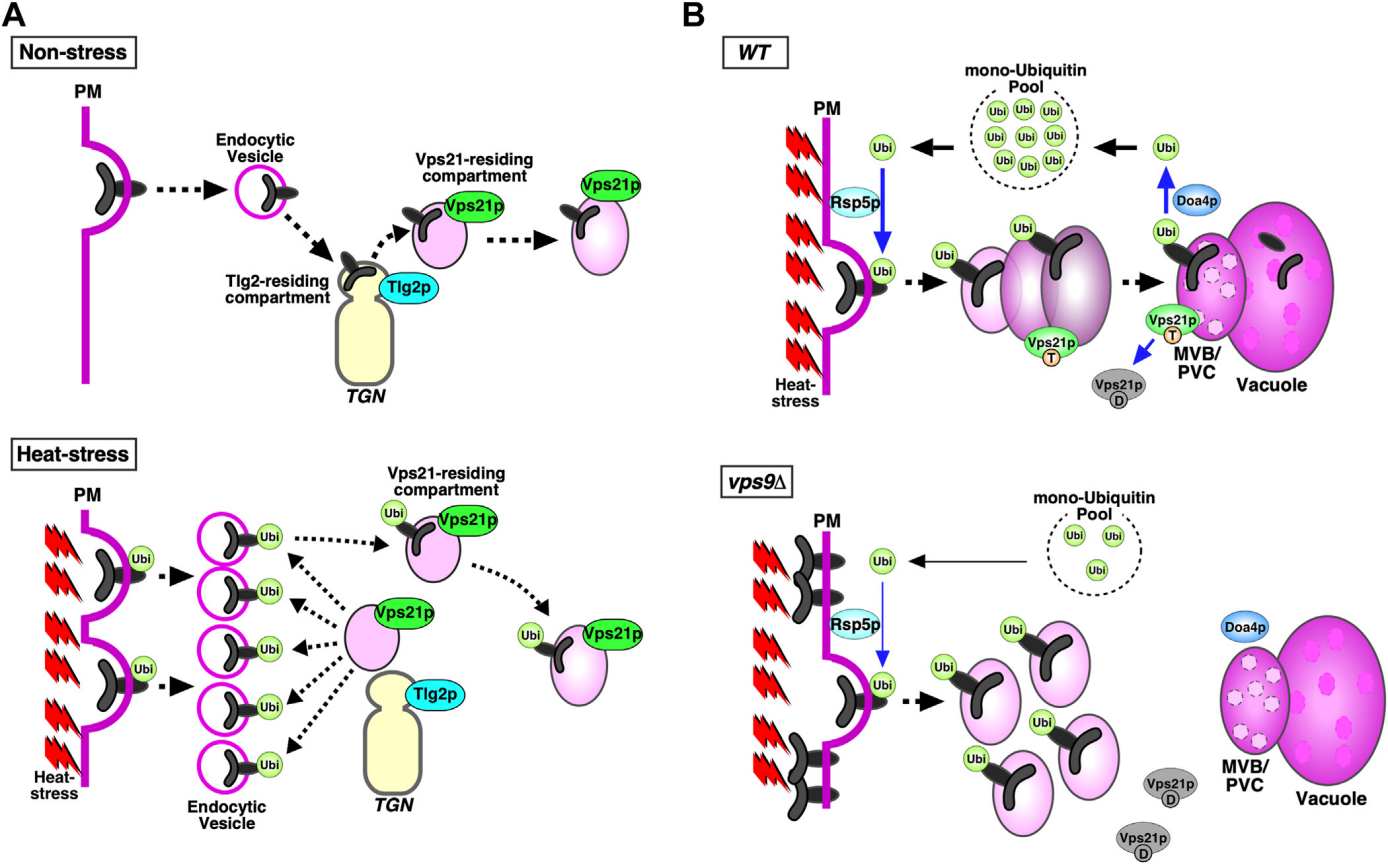
**Model showing the role of Vps21p in ubiquitin-mediated cell surface cargo transport under heat stress conditions in wild-type and *vps9*Δ cells.***A*, model of cargo transport from the plasma membrane (PM) to the Vps21p-enriched compartment under heat stress conditions. Endocytosed cargos are first transported to the Tlg2p-residing compartment within the TGN and then delivered to the Vps21p-enriched compartment. Under heat stress conditions, the Vps21p-enriched compartments change the localization to the PM to facilitate endocytic internalization of the ubiquitinated proteins. *B*, cell surface proteins damaged by heat stress are ubiquitinated by Rsp5p and then internalized from the PM. The endocytosed proteins are transported to the MVB/PVC through Vps21p-enriched endosome, and subsequently degraded within the vacuole. The deubiquitinase Doa4p removes ubiquitin from damaged proteins at the PVC, thereby recycling ubiquitin. In *vps9*Δ cell, transport of ubiquitinated proteins from the PM to the MVB/PVC is impaired, thereby free ubiquitin in the cytosol decreases, causing accumulation of damaged proteins at the PM.

Although Vps21p itself does not have a ubiquitin-binding motif, some interacting proteins, such as Vps9p and Vps15p, have ubiquitin-interacting domains or motifs (34, 35). Furthermore, CORVET subunits Vps8p and Vps11p have RING domains, which are the functional domains of the major E3 ubiquitin ligase family (36, 37). These proteins interacting with Vps21p could act as the sensors for the heat stress-induced misfolding of proteins at the PM, thereby recruiting the Vps21p-enriched compartment to the PM. Recent studies using yeast have demonstrated that endocytosed cargos are first transported to the Tlg2p-residing compartment within the TGN and then delivered to the Vps21p-enriched compartment, dependent on GGA adaptors (7, 38, 39). Thus, surface proteins, including Can1p, damaged by heat stress might also be transported first to the Tlg2p-residing compartment. Consistent with this idea, it has been reported that yeast GGA adaptors are also involved in the maintenance of PM integrity (4). As it has been shown that the Vps21p-enriched compartment interacts with the Tlg2p-compartment (7), PM localization of the Tlg2p-residing compartment might also be increased (Fig. 6*A*).

In cells lacking Vps9p, a Vps21p-specific GEF, PM integrity was impaired and the internalization of Can1p promoted by heat stress was inhibited. Under heat stress conditions deletion of the genes encoding CORVET subunits, Vps3p and Vps8p, or the PtdIns 3-kinase complex subunit Vps15p also resulted in significant impairment of PM integrity, suggesting that deficient endosome maturation leads to defective internalization of aberrant cell surface proteins. In contrast, we and others have shown that deletion of the genes encoding endosomal proteins functioning in the late stage of endosomal trafficking or the formation of MVBs exhibited only a negligible defect of PM integrity (4). As described above, endocytic clearance of cell surface proteins damaged by heat stress is initiated by Rsp5p-ARTs-dependent ubiquitination (4). Doa4p, which is localized at the MVB/late endosome, helps recycle ubiquitin by deubiquitinating cell surface proteins destinated for degradation (24) and thus, in *doa4*Δ cells, the amount of free ubiquitin is markedly reduced (24, 25). Doa4p is reported to localize at an aberrant prevacuolar endosome-like structure in the class E *vps* mutants, including *vps27*Δ and *vps4*Δ cells (40, 41), indicating that endocytic cargo could be deubiquitinated in cells lacking proteins that function in the later stage of endocytosis, thereby maintaining the level of free ubiquitin. Interestingly, we observed that the level of mono-ubiquitin was significantly decreased in cells lacking Vps21p. We also found that overexpression of mono-ubiquitin suppressed the vacuolar localization of GFP-Vps21p and restored Can1p internalization from the PM in *vps9*Δ cells. This might be because highly ubiquitinated endocytic cargos accumulate at immature endosomes in the process of conversion from the early to late endosome and are unable to reach the MBV/late endosome, where the cargos are deubiquitinated by Doa4p (Fig. 6*B*). Similar to the *vps21*Δ mutant, the same class D *vps* mutants, *vps9*Δ, *vps15*Δ, and *vps3*Δ cells, are known to have defective endosome formation and maturation (41). Thus, even in these mutants, ubiquitinated cell surface proteins might accumulate at the immature endosomes, possibly inhibiting the recycling of free ubiquitin and preventing adequate ubiquitination of membrane proteins.

Additionally, in the *doa4*Δ mutant, which shows a decreased level of mono-ubiquitin, we found that Vps21p was mis-transported into the vacuolar lumen, suggesting that Doa4p-mediated deubiquitination seems to play important roles in Vps21 recycling as well as ubiquitin recycling. The mechanism by which Doa4p regulates the localization of Vps21p remains unclear. It has been shown that mammalian Rab5 is monoubiquitinated on three lysine residues (K116, K140, and K165) (42), and monoubiquitination of either K140 or K165 negatively regulates Rab5 activity by altering the intrinsic GDP/GTP conversion cycle in mammals (42). Since K116 and K165 are also conserved in yeast Vps21p, the monoubiquitination of these lysine residues might affect the GTP/GDP binding state, which regulates the localization of Vps21p. Deletion of *DOA4* also resulted in the mis-transport of Vps21p to the vacuolar lumen in cells lacking Vps21p-GAP Gyp3p, indicating that the GTP-bound form of Vps21p is transported into the vacuolar lumen. Although Vps21p is replaced by downstream Rab Ypt7p during the transition from the early to late endosomes *via* the Rab-GEF cascade (43), the precise timing of the Rab5 departure from the endosome is unclear. Our results suggest that the timing of Vps21p detachment from the endosomal membrane might coincide with the deubiquitination of endocytic cargos during the MVB formation. Additionally, the mis-transport of Vps21p into the vacuole might affect Vps21p-dependent Can1p internalization under heat stress conditions. Precisely how deubiquitination of cargos at the MVB/late endosome regulates Vps21p localization remains an important question for the future.

## Experimental procedures

### Yeast strains and plasmids

The yeast strains used in this study are listed in Table S1. All strains were grown in standard rich medium (YPD) or synthetic medium (SM) supplemented with 2% glucose and appropriate amino acids. The NH_2_- or COOH-terminal fluorescent protein tagging of proteins was performed as described previously (8, 44). Fluorescent protein tagging yeast expression constructs were created using pBluescript II (pBS II) vector backbone as previously reported (8, 44).

### Plasma membrane integrity assay

The plasma membrane integrity assay was performed as reported previously (4). In brief, cells were grown to early-to mid-logarithmic phase in SM supplemented with 2% glucose and appropriate amino acids., and then cultured at 40 °C for 3 h ∼1 OD600 cells were pelleted and resuspended in 1 ml PBST (0.01% Tween 20), and stained with 1 μg/ml propidium iodide (Nacalai) for 20 min. After washing twice with PBS, cells were subjected to microscopic analysis.

### Fluorescence microscopy

Fluorescence microscopy was performed using an Olympus IX83 microscope equipped with a x100/NA 1.40 (Olympus) objective and an Orca-R2 cooled CCD camera (Hamamatsu), using Metamorph software (Universal Imaging). For TIRF illumination, an optically pumped semiconductor laser (Coherent) with emission of at 488 nm (OBIS 488LS-50) and at 561 nm (OBIS 561LS-50) were used to excite GFP or mCherry/tdTomato, respectively. Simultaneous imaging of red and green fluorescence was performed using an Olympus IX83 microscope, described above, and an image splitter (Dual-View; Optical Insights) that divided the red and green components of the images with a 565-nm dichroic mirror and passed the red component through a 630/50 nm filter and the green component through a 530/30 nm filter. Dual color time-lapse imaging of red and green fluorescence was performed using an Olympus IX83 microscope equipped with a high-speed filter changer (Lambda 10-3; Shutter Instruments) that can change filter sets within 40 ms. Images for analysis of overlapping localization were acquired using simultaneous imaging (64.5 nm pixel size), described above.

### Image processing and analysis

Overlapping of GFP-Vps21p puncta with Can1-mCherry at the plasma membrane was quantified using the images processed by sequential filtering using the top-hat (16) and bilateral (17) filters bundled in the Image J FIJI software package (18) as described in our recent report (38). Colocalization was defined as occurring when the distance between the two peaks of GFP and mCherry/tdTomato intensities was less than 129 nm (2 pixels).

### Protein extraction from yeast cells

Cells were grown in YPD at 25 °C to OD_600_ of 0.5 to 1.0, harvested by centrifugation, washed with water, and resuspended in lysis buffer (50 mM Tris–HCl, pH 8.0, 150 mM NaCl, 4 M Urea, protease inhibitor cocktail). Glass beads were added to an equal volume and cells were disrupted by Disruptor-Genie (Scientific industry) in the cold room. After incubation with 0.5% Triton X-100 for 10 min, the cleared lysates were diluted with Laemmli’s SDS–PAGE sample buffer.

### Western blot assay

For immunoblot analysis, cell lysates were separated on SDS-PAGE and transferred onto polyvinylidene difluoride membranes (Bio-Rad, Hercules, CA). The membrane was blocked 30 min with 0.5% nonfat dry milk in PBS containing 0.05% Tween 20 and incubated for 2 h at room temperature with anti-ubiquitin antibody (GeneTex, GTX630148) or anti-GAPDH (GeneTex, GTX627408) diluted in Can Get Signal solution 1 (TOYOBO, NKB-201). After washing in PBS containing 0.05% Tween 20, the membrane was incubated for 1 h at room temperature with HRP-linked anti-mouse IgG (GE Healthcare, NA9310) diluted in Can Get Signal solution 2 (TOYOBO, NKB-301). Immunoreactive protein bands were visualized by exposing the membrane for 10 s to 2 min to the WesternLightning Plus ECL reagent (PerkinElmer).

### Statistics

Statistical analysis was performed with GraphPad Prism 7 software, and the data are shown as the mean ± SD or the mean ± S.E.M. as shown in figure legends. Statistical significance was determined using *chi*-square test for trend, unpaired *t* test, or one-way or two-way ANOVA with *post hoc* Turkey’s test as described in the figure legends.

## Data availability

The authors declare that all data supporting the findings of this study are available within the article, its supplementary information files, and/or from the corresponding authors on reasonable request.

## Supporting information

This article contains supporting information.

## Conflict of interest

The authors declare that they have no conflicts of interest with the contents of this article.
