# Supplementary material for: Requirement of Rab5 GTPase during heat stress-induced endocytosis in yeast
Source: J Biol Chem. 2024 Jul 11;300(8):107553. doi: 10.1016/j.jbc.2024.107553 (PMC11345375; doi:10.1016/j.jbc.2024.107553)
Supplement: Supporting Table [file mmc1.docx]

**Supplementary Table1**. Yeast Strains used in this study

Strain Genotype Source

JJTY1219 *Mat***a** *his3*Δ*1* *leu2*Δ*0* *ura3*Δ*0* *lys2*Δ*0* Toshima lab

JJTY3638 *Mat***a** *his3*Δ*1* *leu2*Δ*0* *ura3*Δ*0* *lys2*Δ*0 GFP-VPS21*::*HIS3* Toshima lab

JJTY5511 *Mat***a** *his3*Δ*1* *leu2*Δ*0* *ura3*Δ*0* *lys2*Δ*0 GFP-VPS21*::*HIS3 HSE1-tdTomato*::*URA3* Toshima lab

JJTY5227 *Mat***a** *his3*Δ*1* *leu2*Δ*0* *ura3*Δ*0* *lys2*Δ*0 GFP-VPS21*::*HIS3 VPH1-mCherry*::*URA3* Toshima lab

YKO No.1-E-3 *Mat***a** *his3*Δ*1 leu2*Δ*0 ura3*Δ*0 met15*Δ*0 vps8*Δ::*KanMX6* Dharmacon^TM^

YKO No.7-A-2 *Mat***a** *his3*Δ*1 leu2*Δ*0 ura3*Δ*0 met15*Δ*0 vps21*Δ::*KanMX6* Dharmacon^TM^

YKO No.11-A-2 *Mat***a** *his3*Δ*1 leu2*Δ*0 ura3*Δ*0 met15*Δ*0 doa4*Δ::*KanMX6* Dharmacon^TM^

YKO No.26-A-7 *Mat***a** *his3*Δ*1 leu2*Δ*0 ura3*Δ*0 met15*Δ*0 vps4*Δ::*KanMX6* Dharmacon^TM^

YKO No.30-E-3 *Mat***a** *his3*Δ*1 leu2*Δ*0 ura3*Δ*0 met15*Δ*0 vac1*Δ::*KanMX6* Dharmacon^TM^

YKO No.34-C-3 *Mat***a** *his3*Δ*1 leu2*Δ*0 ura3*Δ*0 met15*Δ*0 vps9*Δ::*KanMX6* Dharmacon^TM^

YKO No.40-D-6 *Mat***a** *his3*Δ*1 leu2*Δ*0 ura3*Δ*0 met15*Δ*0 vps27*Δ::*KanMX6* Dharmacon^TM^

YKO No.70-C-5 *Mat***a** *his3*Δ*1 leu2*Δ*0 ura3*Δ*0 met15*Δ*0 vps45*Δ::*KanMX6* Dharmacon^TM^

YKO No.70-H-3 *Mat***a** *his3*Δ*1 leu2*Δ*0 ura3*Δ*0 met15*Δ*0 vps15*Δ::*KanMX6* Dharmacon^TM^

YKO No.48-E-2 *Mat***a** *his3*Δ*1 leu2*Δ*0 ura3*Δ*0 met15*Δ*0 vps3*Δ::*KanMX6* Dharmacon^TM^

JJTY2702 *Mat***a** *his3*Δ*1* *leu2*Δ*0* *ura3*Δ*0* *lys2*Δ*0 vps27*Δ::*KanMX6 GFP-VPS21*::*HIS3* This study

JJTY4545 *Mat***a** *his3*Δ*1* *leu2*Δ*0* *ura3*Δ*0* *lys2*Δ*0 vps4*Δ::*KanMX6 GFP-VPS21*::*HIS3* This study

JJTY4558 *Mat***a** *his3*Δ*1* *leu2*Δ*0* *ura3*Δ*0* *lys2*Δ*0 bro1*Δ::*KanMX6 GFP-VPS21*::*HIS3* This study

JJTY5228 *Mat***a** *his3*Δ*1 leu2*Δ*0 ura3*Δ*0 met15*Δ*0 gyp3*Δ::*KanMX6 GFP-VPS21*::*HIS3 VPH1-mCherry*::*URA3* This study

JJTY9772 *Mat***a** *his3*Δ*1 leu2*Δ*0 ura3*Δ*0 lys2*Δ*0* *CAN1-GFP*::*HIS3* This study

JJTY9773 *Mat***a** *his3*Δ*1 leu2*Δ*0 ura3*Δ*0 met15*Δ*0 vps9*Δ::*KanMX6 CAN1-GFP*::*HIS3* This study

JJTY9878 *Mat***a** *his3*Δ*1 leu2*Δ*0 ura3*Δ*0 met15*Δ*0 art1*Δ::*KanMX6 art2*Δ::*LEU2* This study

JJTY11617 *Mat***a** *his3*Δ*1 leu2*Δ*0 ura3*Δ*0 met15*Δ*0 doa4*Δ::*KanMX6 CAN1-GFP*::*HIS3* This study

RRS32 *Mat***a** *his3*Δ*1 leu2*Δ*0 ura3*Δ*0 met15*Δ*0 doa4*Δ::*KanMX6 GFP-VPS21*::*HIS3* This study

RRS84 *Mat***a** *his3*Δ*1 leu2*Δ*0 ura3*Δ*0 met15*Δ*0 doa4*Δ::*KanMX6 gyp3*Δ::*LEU2 GFP-VPS21*::*HIS3 VPH1-mCherry*::*URA3* This study

RRS90 *Mat***a** *his3*Δ*1 leu2*Δ*0 ura3*Δ*0 lys2*Δ*0* *GFP-VPS21*::*HIS3 [pUB175 {pRS426-myc-Ubiquitin (CUP1 promoter)}]* This study

RRS91 *Mat***a** *his3*Δ*1 leu2*Δ*0 ura3*Δ*0 met15*Δ*0 doa4*Δ::*KanMX6* *GFP-VPS21*::*HIS3*

*[pUB175 {pRS426-myc-Ubiquitin (CUP1 promoter)}]* This study

RRS629 *Mat***a** *his3*Δ*1 leu2*Δ*0 ura3*Δ*0 lys2*Δ*0* *GFP-VPS21*::*HIS3 CAN1-mCherry*::*URA3* This study
