# Supplementary material for: Requirement of Rab5 GTPase during heat stress-induced endocytosis in yeast
Source: J Biol Chem. 2024 Jul 11;300(8):107553. doi: 10.1016/j.jbc.2024.107553 (PMC11345375; doi:10.1016/j.jbc.2024.107553)
Supplement: Supporting Information [file mmc2.pdf]

**Supplementary Information**

# Requirement of Rab5 GTPase during heat stress-induced endocytosis in yeast

**Authors**

Makoto Nagano[1,2]*, Hiroki Shimamura[1], Junko Y. Toshima[3], and Jiro Toshima[1]*

**Affiliations**

[1]Department of Biological Science and Technology, Tokyo University of Science, 6-3-1 Niijyuku, Katsushika-ku, Tokyo 125-8585, Japan

[2]Research Center for Drug and Vaccine Development, National Institute of Infectious Diseases, 1-23-1 Toyama, Shinjuku-ku, Tokyo 162-8640, Japan

[3]School of Health Science, Tokyo University of Technology, 5-23-22 Nishikamata, Ota-ku, Tokyo 144-8535, Japan

## Supplementary Figure legends

**Figure S1. Requirement of endosome-related proteins for maintaining the plasma membrane integrity under heat stress condition.** (**A** and **C**) Propidium iodide (PI) staining of the mutant cells under heat stress condition. The cells were analyzed in the same way as Figure 1B. Fluorescence images overlaid with DIC images were shown in the left panels. For quantifying the percentage of PI-positive cells, fluorescence images were converted to the binary images as shown in right panels. (**B**) The percentage of PI-

positive cells in the mutant cells. Data show the mean ± SEM of three independent experiments in which 100 cells were scored per experiment (B). Different letters indicate significant difference at $p < 0.05$, one-way ANOVA with Tukey's post-hoc test (B). Scale bar in all panels, 2.5 μm

**Figure S2. Heat stress or Arginine-induced internalization of the arginine transporter Can1p.** (**A**) Localization of Can1-GFP in wild-type or mutant cells under heat stress condition. Cells were grown to early-mid logarithmic phase at 25°C, then cultured at 40°C for 40 min. Higher magnification view of the boxed area in the upper images are displayed in the middle panels. (**B**) Quantification of Can1-GFP localization in wild-type or mutant cells after being cultured at 40°C for 40 min. Plasma membrane only (PM), plasma membrane and endosome (PM + End), and plasma membrane, endosome and vacuole (PM + End + Vac). (**C**) Localization of Can1-GFP in wild-type cells under heat stress condition in the presence or absence of 10% glycerol. Cells were grown to early-mid logarithmic phase at 25°C, cultured with 10% glycerol for 1 hr, then shifted to 40°C for 40 min. Representative fluorescence intensity profiles along the green line in the upper images are indicated in the lower images. (**D**) Localization of Can1-mCherry and GFP-Vps21p at the cell surface under heat stress condition in the presence of 10% glycerol. Cells expressing Can1-mCherry and GFP-Vps21p were grown to early-mid logarithmic phase at 25°C, cultured with 10% glycerol for 1 hr, shifted to 40°C for 40 min, and analyzed by TIRF microscopy. The fluorescent images (Raw image) were processed to visualize the punctate localization of Can1-mCherry and GFP-Vps21p at the

cell surface (Processed image) by sequential filtering using top-hat and bilateral filters bundled in the Image J FIJI software package as described in the Materials and Methods section. Higher magnification view of the boxed area in the processed images are displayed in the lower panels. (E) Quantification of the number of GFP-Vps21p puncta at 40°C in the presence or absence of 10% glycerol. (**F**) Localization of Can1-GFP in wild-type or *vps9*Δ cells in the presence or absence of 5mM Arginine. Cells were grown to early-mid logarithmic phase at 25°C, and cultured for 30 min in the presence or absence of 5mM Arginine. (**G**) Quantification of Can1-GFP localization in wild-type or *vps9*Δ cells in the presence of 5mM Arginine. Plasma membrane only (PM), plasma membrane and endosome (PM + End), and plasma membrane, endosome and vacuole (PM + End + Vac). Data show the mean ± SD with 50 puncta (E) from three independent experiments or the mean ± SEM of three independent experiments in which 100 cells (B, G) were scored per experiment. ****$p < 0.0001$, *chi*-square test for trend (B, G), two-tailed unpaired *t*-test with Welch's correction (E). Scale bar in panels in A, C, D (upper) and F, 2.5 μm, or D (lower), 1.0 μm

**Figure S3. Effect of proteotoxic stress on the Vps21p localization and role of TGN-resident factors in the heat-stress induced formation of Vps21p-enriched compartment.** (**A**) Maximum-intensity projections of z-stacks of the cells expressing GFP-Vps21p. Cells expressing GFP-Vps21p were grown to early-mid logarithmic phase at 25°C, then cultured at 40°C for the indicated time. Green and yellow arrows indicate the localization of Can1-GFP at endosome-like compartments and in vacuole (upper

panels). Magenta arrows indicate that Can1-GFP resides at the plasma membrane even after heat stress (lower panels). grown to early-mid logarithmic phase at 25°C, then cultured in the presence of 5 mM dithiothreitol (DTT) or 10% ethanol (EtOH) for 2 hour, and analyzed by epifluorescence microscopy. The z series was acquired through the entire cell at 0.4 μm intervals. (**B**) Localization of Can1-mCherry and GFP-Vps21p in the presence of DTT or EtOH. Cells expressing GFP-Vps21p and Can1-mCherry (Can1-mCH) were grown to early-mid logarithmic phase at 25°C, then cultured in the presence of 5 mM DTT or 10% EtOH for 2 hour, and analyzed by epifluorescence microscopy. Green and yellow arrows indicate the localization of Can1-mCH puncta overlapping with GFP-Vps21p at the PVC and Can1-mCH in the vacuolar lumen. (**C**) The spatio-temporal localization of GFP-Vps21p in wild-type or mutant cells under heat stress condition. Cells were grown to early-mid logarithmic phase at 25°C, then cultured at 40°C for the indicated time, and analyzed by epifluorescence microscopy. (**D**) Quantification of the number of GFP-Vps21p puncta in the wild-type or mutant cells under heat stress condition. Data show the mean ± SD with 150 puncta (D) from three independent experiments. ****$p$ <0.0001, one-way ANOVA with Tukey's post-hoc test (C). Scale bar in all panels, 2.5 μm

**Figure S4. Effect of *DOA4* on localization of Vps21p.** (**A**) Localization of GFP-Vps21p in wild-type and mutant cells under non-stress condition. Yellow and red arrows indicate the localization of GFP-Vps21p at the PVC and the vacuolar lumen. (**B**) Localization of GFP-vps21(Q66L) mutant in *doa4*Δ cells. Green and yellow arrows indicate the

localization of GFP-Vps21p at the vacuolar membrane and PVC. Red arrow indicates the localization in the vacuolar lumen under heat stress condition. Scale bar in all panels, 2.5 μm

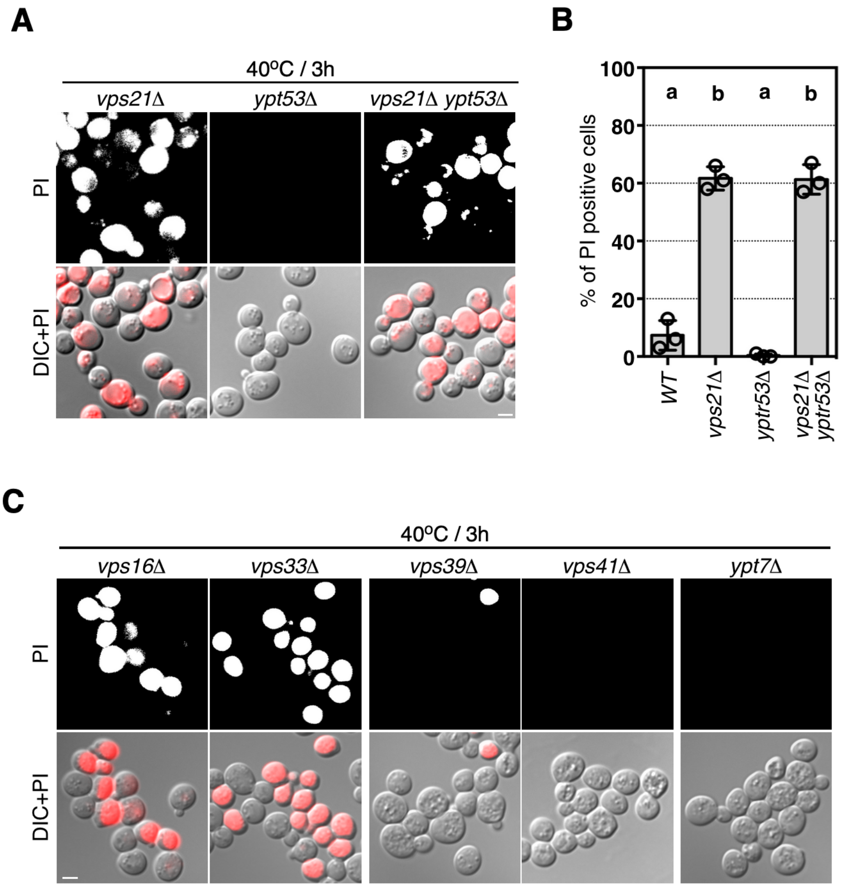


**Figure S1. Nagano et al.**

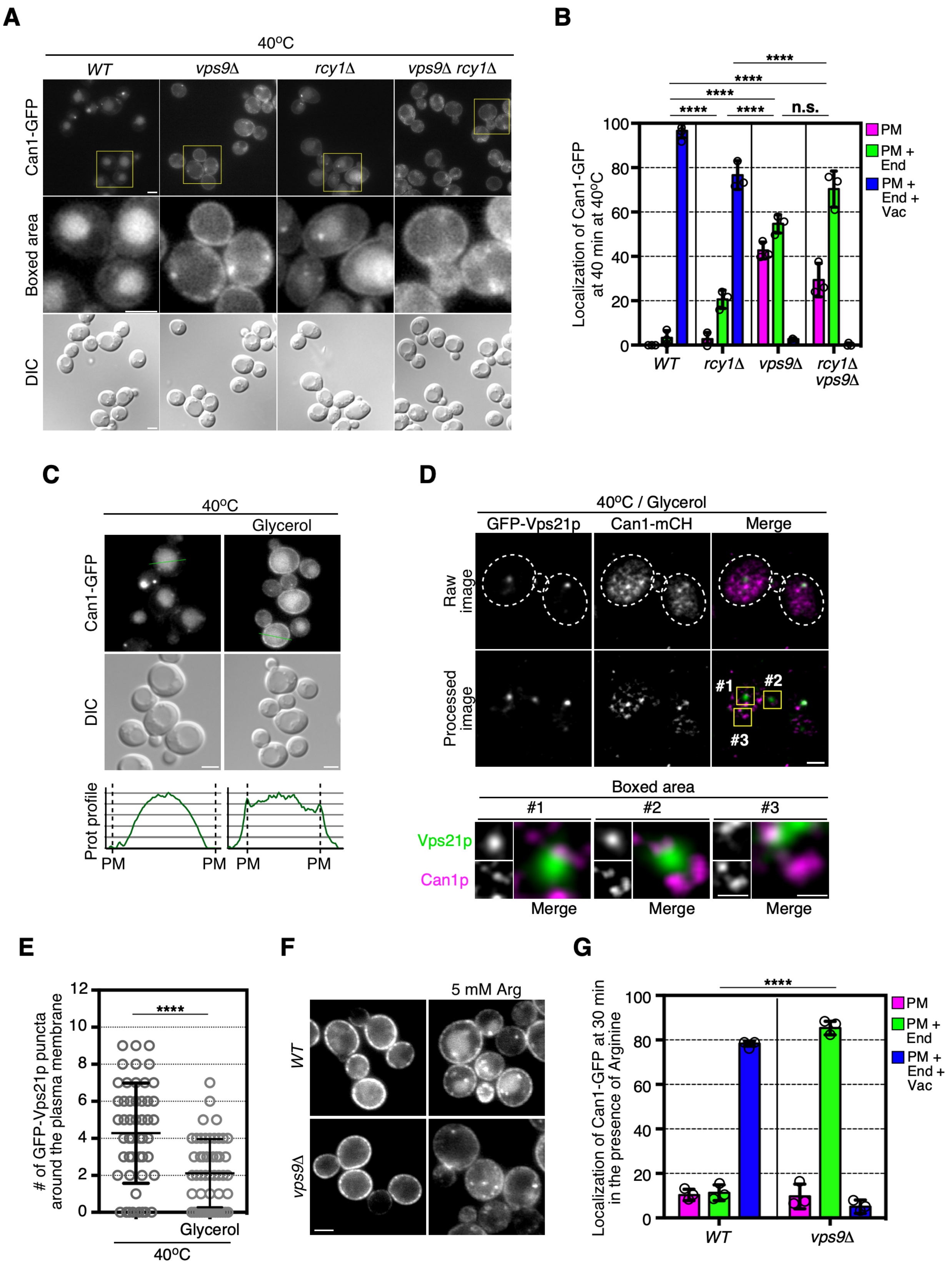


**Figure S2. Nagano et al.**

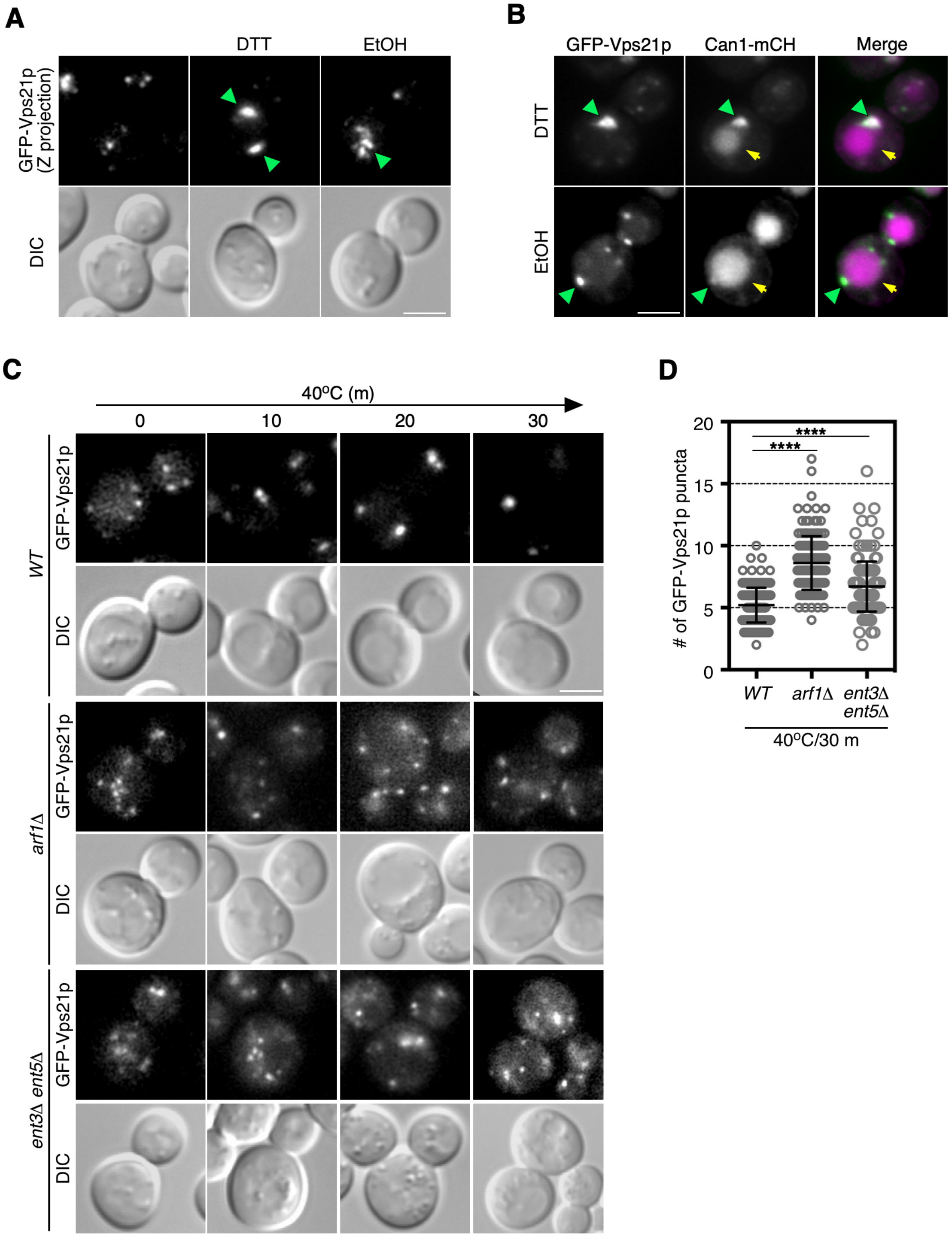


**Figure S3. Nagano et al.**

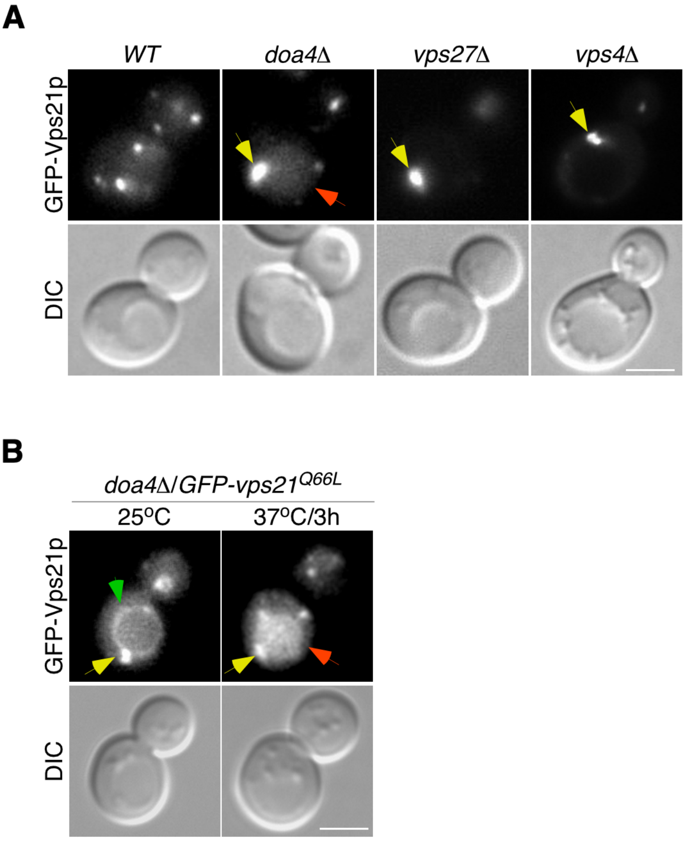


**Figure S4. Nagano et al.**
